# Electronic Medication Reconciliation Tools Aimed at Healthcare Professionals to Support Medication Reconciliation: a Systematic Review

**DOI:** 10.1007/s10916-023-02008-0

**Published:** 2023-12-06

**Authors:** Pablo Ciudad-Gutiérrez, Paula del Valle-Moreno, Santiago José Lora-Escobar, Ana Belén Guisado-Gil, Eva Rocío Alfaro-Lara

**Affiliations:** Department of Pharmacy, University Hospital Virgen del Rocio, Av. Manuel Siurot s/n., 41013 Seville, Spain

**Keywords:** Healthcare professionals, Medication reconciliation, Patient safety, Electronic tools

## Abstract

The development of health information technology available and accessible to professionals is increasing in the last few years. However, a low number of electronic health tools included some kind of information about medication reconciliation. To identify all the electronic medication reconciliation tools aimed at healthcare professionals and summarize their main features, availability, and clinical impact on patient safety. A systematic review of studies that included a description of an electronic medication reconciliation tool (web-based or mobile app) aimed at healthcare professionals was conducted. The review protocol was registered with PROSPERO: registration number CRD42022366662, and followed PRISMA guidelines. The literature search was performed using four healthcare databases: PubMed, EMBASE, Cochrane Library, and Scopus with no language or publication date restrictions. We identified a total of 1227 articles, of which only 12 met the inclusion criteria.Through these articles,12 electronic tools were detected. Viewing and comparing different medication lists and grouping medications into multiple categories were some of the more recurring features of the tools. With respect to the clinical impact on patient safety, a reduction in adverse drug events or medication discrepancies was detected in up to four tools, but no significant differences in emergency room visits or hospital readmissions were found. 12 e-MedRec tools aimed at health professionals have been developed to date but none was designed as a mobile app. The main features that healthcare professionals requested to be included in e-MedRec tools were interoperability, “user-friendly” information, and integration with the ordering process.

**Supplementary Information:**

The online version contains supplementary material available at 10.1007/s10916-023-02008-0.

## Introduction

Medication reconciliation (MedRec) is a recognized strategy to improve patient safety during transitions of care. It consists of establishing an accurate list of all the medications that a patient is actually taking to provide correct medications to patients and prevent adverse drug events [1]. MedRec is a complex and challenging process that requires the time and collaboration of all involved healthcare professionals to carry out the appropriate medication changes and communicate them properly to patients [2].

International patient safety organizations agree on the necessity of implementing MedRec during care transitions as an effective strategy in patient care and promoting the use of information technologies (IT) as a support tool for this procedure [3, 4]. Paper-based systems are known to take more time than electronic MedRec (e-MedRec) tools [5]. The benefits of using IT for MedRec are not only workflow optimization but also the greater ability to integrate pharmacotherapeutic information and achieve better results in detecting medication discrepancies [5, 6].

Previous studies showed that IT in healthcare systems was mainly focused on collecting medication information, and very few institutions had already incorporated an e-MedRec tool in their routine clinical practice [7]. In this way, a recent study showed that, despite the increase in the number of mobile health applications (apps), widely available and accessible for professionals, there is still a lack of apps that include information about MedRec [8]. In contrast, computerized tools have a greater presence and usability in this field, including promising results in health outcomes [9, 10].In fact, previous publications have shown a reduction of 45% in drug omissions using an e-tool and reductions of unintended discrepancies with the use of IT [5, 11]. A previous review, published in 2017, summarized websites or software up to October 2014. However, although the tools scored well in terms of user adherence, satisfaction, and usability, only English-language tools were examined and additional details were lacking [9]. In this sense, more extensive research on this topic could provide an update on available e-MedRec tools and collect more data on their design, development, and effectiveness.

In the last few years, we have all witnessed an exponential increase in the use of technology in different healthcare settings probably foster by the emergence and subsequent pandemic of the coronavirus disease 2019 (COVID-19) [12]. Patients and their caregivers have been the focus of IT, so there have been few studies on the use of telehealth and digital technology by healthcare professionals. Thus, the objective of the study was to identify the existing e-MedRec tools (web-based or mobile apps) aimed at healthcare professionals and to summarize their main characteristics, availability, and clinical impact on patient safety.

## Methods

### Information sources and search strategy

An electronic literature search was performed using four Healthcare Databases: PubMed, EMBASE, Cochrane Library, and SCOPUS, with no language or publication date restrictions up to 15 December 2022. Search terms included a mixture of MeSH terms and free text (keywords, synonyms, and word variations) combined with Boolean operators. The search strategy is detailed in Table S1 from Supplementary File 1. Authors were contacted for further information in the absence of sufficient data. The reference lists of selected studies were also hand-searched to identify any other relevant studies that evaluated and provided more information about the e-tools detected.

### Eligibility criteria

This systematic review was carried out in accordance with the Preferred Reporting Items for Systematic Reviews and Meta-Analyses (PRISMA) guidelines [13]. The completed PRISMA checklists are included as Supplementary File 2. The review protocol was registered in the International Prospective Register of Systematic Reviews (PROSPERO) Database (registration number:CRD42022366662). The selected studies were those that met the following inclusion criteria:

- The study included a description of the e-MedRec tool (web-based or mobile app) aimed at healthcare professionals.
- The e-MedRec tool could be used for MedRec of pediatric or adult patients.
- The e-MedRec tool could be used at any point of care transition (admission, discharge, or in outpatient clinics).
- No language restrictions.

We excluded:

- Studies describing electronic tools for purposes other than MedRec.
- Studies referring to a description of a non-electronic MedRec tool.
- The e-MedRec tool was designed to be used by patients exclusively.
- Studies that were not available in full text or abstract.

### Study selection

The titles and abstracts of all eligible articles were screened for inclusion by two independent reviewers (PCG and PVM). Any disagreements were settled by consensus or with a third reviewer (ABGG). Finally, we evaluated the considered full-length publications before a final decision on inclusion was reached by all reviewers.

### Data collection and quality assessment

The data extraction guide was created according to the recommendations of some authors on the minimum content required to adequately describe e-MedRec tools and user perceptions [9, 11]. Two reviewers (PCG and PVM) independently extracted data from the papers and grouped the records as those that specifically focused on the description of each study or the selected e-tools. ABGG checked all extraction sheets for accuracy and resolved any discrepancies by independent review of the full-length publications. We explicitly stated if there were any missing data from studies. For each publication, the following variables were registered:

- Author, country, and year of publication.
- Name of e-MedRec tool detected.
- The objective of the study.
- Study design: randomized clinical trial (RCT), quality improvement project (QIP), or observational study.
- Eligible patients for the study.
- Transition points of care involved:admission, discharge,and outpatient setting.

Moreover, these variables were recorded for each tool:

- Entry of patient data and medication information into the tool: automated or manual.
- Availability of the tool: mobile app, website, or software.
- Clinical impact on patient safety: reduction of medication errors, discrepancies, adverse drug events, emergency visits, or hospital readmissions.
- Users (healthcare professionals): pharmacists, nurses, and/or physicians.
- Features related to the ease of use and comprehension of e-tools: displaying different medication lists, transferring information between healthcare professionals, grouping medications, generating a reconciliation report, color coding, or triggering MedRec alerts.
- Opinions of the users about usability, adherence, satisfaction, and their recommendations to improve the e-tools.

A quality assessmentof each report was carried out in accordance with the study design. QIPs were evaluated by the Standards for Quality Improvement Reporting Excellence (SQUIRE) guidelines [14], RCTs by the Consolidated Standards of Reporting Trials (CONSORT) criteria [15] and the Strengthening the Reporting of Observational Studies in Epidemiology (STROBE) statement was used for reporting observational studies [16].

In addition, a multidimensional framework was used to assess the quality of each e-tool [17]. This framework aggregated social, technical, and organizational evaluation criteria that were considered essential for the design and development of a sophisticated e-health tool. According to these criteria, variables related to the context, user perception or implementation of the tool were recorded. However, those functionalities of the reported tools that were poorly evaluated by the authors or were irrelevant to the optimization of future e-MedRec tools were not included in this work.

## Results

**Fig. 1:**
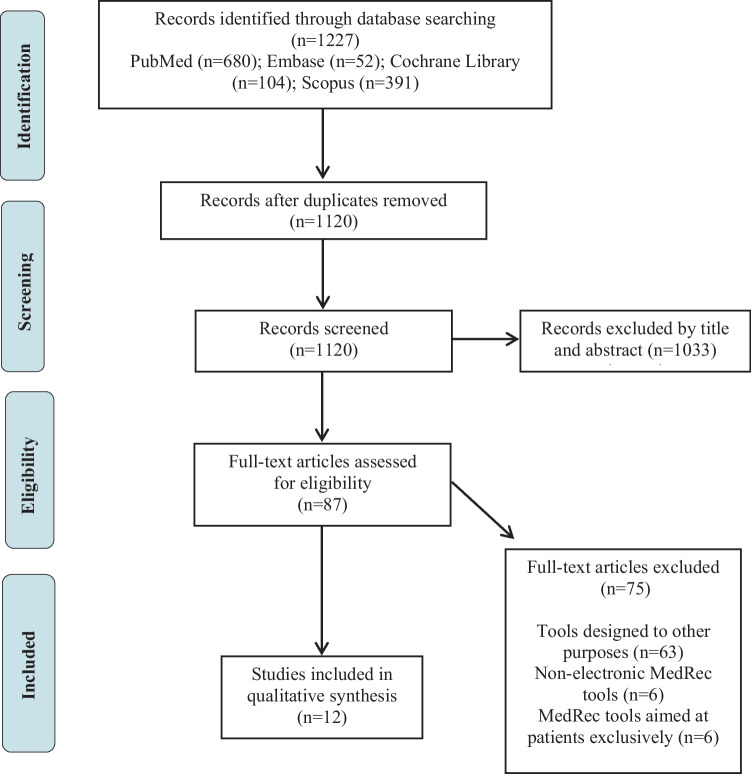
Study selection flowchart. MedRec, medication reconciliation

1227 articles were identified through database searching. After the elimination of duplicates, 1120 were collected by title and abstract. 87 records were assessed for eligibility, but only 12 met the inclusion criteria (Fig. 1) [18–29].From the reference lists, six relevant studies that evaluated and provided more information about four of the e-MedRec tools were identified [30–35].

A quality assessment of the 12 selected studies was carried out. For QIPs, there was wide heterogeneity in the reporting criteria between studies, with some aspects of the available evidence or the purpose of the project being well documented, although ethical considerations were lacking in the majority of the studies. For RCTs, the items with the highest reporting rate (> 90%) were: title and abstract, background and eligibility criteria for participants, while the least covered standards (< 10%) were: changes to trial outcomes after the start of the trial or performing subgroup analyses. Finally, the observational study met most of the criteria, but potential sources of bias were not addressed.

The main characteristics of the studies which were included in the systematic review are summarized in Table 1. Six of the 12 studies were published over the last 10 years and two after 2020. Most studies were carried out in the USA (8), two were in Canada and only one in Belgium and Spain. With respect to study design, 8 were QIPs, 3 RCTs and only one was an observational study. Most transition points of care investigated were admission and discharge (2), discharge (4), outpatients (4) one in admission, transfer, and discharge (1), and the remaining one in admission (1). Four studies had no restrictions on eligible patients and no data were found in three. Test patients were included in one study [28].

**Table 1:** Description of the functionalities of the e-tools and their clinical impact on reconciliation

**Author, year**	**Country**	**e-MedRec tool**	**Objective**	**Study design**	**Eligible patients for the study**	**Transition points of care investigated**	**Entry of patient data and medication information**	**Availability**	**Clinical impact on patient safety**
Poon et al., 2006 [18]	USA	The PAML Builder	To design and develop an e-MedRec tool-supported of the MedRec that combined multiple sources of patient information	QIP	No restrictions	Admission, discharge	Automatically (from EMR and CPOE systems) and manually	Software	Reduction of potential ADEs detected per patient(1.44 in the control arm and 1.05 in the intervention arm)^32^
Vawdrey et al., 2010 [19]	USA	A MedRec View	To assess the impact of using the e-MedRec tool with hard stop reminders during the MedRec process	QIP	No restrictions	Admission, discharge	Manually	Software	-
Giménez-Manzorro et al., 2011 [20]	Spain	Aplicon	To evaluate the impact of the tool in minimizing unintended discrepancies among medication patient lists before and after a surgery	QIP	No restrictions	Admission	Manually	Software	Reduction in the percentage of unintended discrepancies (from 3.5% to 1.8%)^33^ Reduction in the percentage of unintendeddiscrepancies (from 10.6% to 6.6%)^34^
Schnipper et al., 2011 [21]	USA	The Partners PostDischarge Medication Reconciliation Tool	To describe the design and implementation of the tool by ambulatory clinicians after hospital discharge	O	No restrictions	Discharge	Manually	Software	-
Lovins et al., 2011 [22]	USA	Electronic pathway for MedRec	To assess the reduction of medication errors of a novel application after certain transitions of care	QIP	-	Admission, transfer and discharge	-	Software	-
Tamblyn et al., 2012 [23]	Canada	Electronical enabled discharge reconciliation tool	To evaluate if an e-MedRec application reduced the risk of adverse drug events, hospital readmissions and emergency visits	RCT	18 years or older patients admitted to a general medical or surgical unit or intensive care and discharged alive	Discharge	Automatically (retrieval of community drug lists from community pharmacy records)	Software	-
Cadwallader et al., 2013 [24]	USA	MedRec user interface	To design an e-MedRec tool that evaluated the patient adherence to their medications	QIP	-	Outpatients	-	Software	-
Plaisant et al., 2013 [25]	USA	Twinlist	To describe a novel interface to facilitate the clinician workflow during MedRec process	QIP	-	Discharge	Manually	Open-source website available at: http://www.cs.umd.edu/hcil/sharp/twinlist	Reduction in the number of critical medication errors (24 in the control group and 7 with Twinlist) and serious medications errors (8 in the control group and 4 with Twinlist) were detected^35^
Tamblyn et al., 2018 [26]	Canada	The RightRx Project	To assess the impact of a new e-MedRec tool to reduce clinician workload	RCT	Patients in medical and surgical units	Discharge	Automatically (retrieval of community drug lists from community pharmacy records)	Software	Reduction in medication discrepancies among the intervention group (26.4%) compared with usual care (56.0%)^36^ No significant differences were found between the emergency room visits (1.4 vs 1.2) and hospital readmissions (0.3 vs 0.2) among the intervention group and usual care respectively^36^
Marien et al., 2019 [27]	Belgium	MedRec web-app	To evaluate the usability of the application by users' opinions, reporting the results about the low, medium and high prototypes	QIP	Volunteers	Outpatients	Automatically (from the Regional eHealth Network)	Website	-
Co et al., 2021 [28]	USA	The Ambulatory electronic health record evaluation tool	To describe the development of a new e-MedRec tool and reporting their qualitative and quantitative results in medication safety and medication reconciliation	QIP	Only test patients were used	Outpatients	Manually	Software	-
Gionfriddo et al., 2022 [29]	USA	MedTrue	To analyze the effectiveness and satisfaction of a web-based application by patients and clinical staff	RCT	18 years of age or older patients, able to speak English, and seen at a participating site by a member of the primary care team	Outpatients	Automatically (from EMR)	Software	No differences in medication discrepancies and medication list accuracy between the intervention group and usual care

*PAML* Preadmission Medication List, *MedRec* Medication Reconciliation, *EMR* Electronic medical record, *USA* United States of America, *QIP* Quality improvement project, *O* observational study, *RCT* Randomized clinical trial. Quality improvement projectsinvolved systematic, data-guided initiatives or processes designed to improve clinical care, patient safety, health care operations, services and programs or for developing new programs or services, *CPOE* Computerized provider order entry, *ADEs* adverse drug events

Table 1 also shows the description of e-MedRec tools including the data entry, availability, and clinical impact on patient safety. Four e-tools [23, 26, 27, 29] could retrieve automatically information related to patients and their medication from community-drug lists, electronic medical records (EMR), or computerized provider order entry systems. One e-tool [18] allowed users to manually introduce the patient medication list or retrieve the last updated prescription from multiple electronic sources. Concerning the availability of the e-MedRec tools, all of them were computerized (software [18–24, 26, 28, 29] or website [25, 27]. “MedRec view” [19] was a commercially available software. Some of the software were embedded throughout EMR products such as Eclipsys Corporation [19], Siemens [22], Leapfrog [28], and EpicCare [29].Two tools were developed and provided by “Partners HealthCare System” [18, 20]. Two tools [20, 27] were created as a software prototype to incorporate into the EMR.“Twinlist” [19] was the only open-source website. The “MedRec web-app” [27] was a web application link available for all clinicians who have access to the Regional eHealth network with a mandatory log-in and secure password. No e-MedRec tool was implemented as a mobile app, but, clinicians who used the “MedRec web-app” [27] were interested in the adoption of the e-tool to be usable on smartphones and tablets. For the clinical impact of e-tools, some of them showed a reduction of medication errors, discrepancies, and adverse drug events among intervention patients compared to the usual care. However, no significant differences in emergency room visits or hospital readmissions were found with the use of “The RightRx Project” [26, 35].

Different functionalities were implemented in the e-tools to make the MedRec process more productive. Some examples are: displaying and comparing different medication lists ordered in columns to easily identify medication errors [19, 24–27, 29], transferring information between healthcare professionals [20, 26, 27], or grouping medicationsin different categories (therapeutic class, diagnosis, dosage, or ordered by clinical importance) [18, 25, 27]. Additional features were giving information to users about drug allergies or drug interactions [23] or clicking a button to continue, change or stop a medication [24, 26, 27]. Only the¨RightRx Project” [26] was able to generate a conciliation report that can be printed and given to patients once the medication list was updated. This tool was also the first to incorporate a data warehouse with a register of patient medication, prescribers, and dispensing pharmacies into the app. Additionally, a summary of the reconciled medication list in a “patient-friendly” language that could be printed and given to the patients might be included in the next prototypes of the “MedRec web-app”. Moreover, the authors of this application tested the opinion of users at various stages to obtain a sophisticated tool as a final product [27]. Color codes were implemented in the “Twinlist” [25] and “MedRec web-app” [27] to facilitate the MedRec process by healthcare professionals. Moreover, four tools were capable of triggering alerts or reminders to the providers related to medication discrepancies in order to enhance patient safety [19, 21, 24, 28].

The users' quality assessment of e-MedRec tools was evaluated in Table 2. Firstly, the majority of tools were aimed at physicians and pharmacists (6) while others could be used by physicians, pharmacists and nurses (3), physicians (2), and the remaining one was aimed at physicians, pharmacists, nurses, and patients. With respect to adherence, some authors measured the percentage of clinicians who used the e-tools [19, 21, 22, 27, 29] but others showed the number of medication lists updated by clinicians among patients [28]. Usability was measured by the reduction in time of MedRec process using the tool [18, 19, 25, 26], improvement in clinician workflow [22, 24], or patient safety [22, 28]. Clinician surveys were mainly used to assess satisfaction with the tools [18, 22, 29]. Finally, most of the users’ suggestions were aimed at the integration of the e-tool with the ordering process [18, 19, 27], interoperability [19, 27], and easier ways to reconcile the medication list [18, 19, 21, 26, 29].

**Table 2:** Users' quality assessment of e-MedRec tools

**e-MedRec tool**	**Users**	**Adherence**	**Usability**	**Satisfaction**	**Users suggestions**
The PAML Builder [19, 31, 32]	Pharmacists, nurses and physicians	-	Improvement in patient care creating a PAML according to 64% of responders Reduction in time to carry out MedRec (< 10 min)	39% were satisfied and 32.1% reported neutrality	Ease of adding new medications (medication specific-doses or frequencies) Integration with the ordering process (transference of the PAML to the order entry)
A MedRec view [20]	Pharmacists, nurses and physicians	Low (< 40%) increased up to 96% after a reminder intervention	Reduction in time from 84.5 h up to 9.2 h between hospital admissions and MedRec	-	Easier ways to add new medications (30–60 s per medication in the reported tool) Integration with the ordering process must be better addressed in future prototypes Improvement in user interfaces with dispensing pharmacies and personal health records
Aplicon [21, 33, 34]	Physicians and pharmacists	-	Patient safety (the tool alerts clinicians to review all the changes made on the patient medication list)	Staff nurses revealed how tough was to record patient medication in the tool	-
The Partners PostDischarge Medication Reconciliation Tool [22]	Primary-care physicians	Low (20%), increased up to 41% after a reminder intervention	-	Accepted by most participants in theory based on survey results	Increasing the applicability of the tool for more type of patients (only available for outpatients)
Electronic pathwayfor MedRec [23]	Physicians	Compliance rate > 90%	Improvement in workflow, efficiency and patient safety	Great satisfaction	-
Electronical enabled discharge reconciliation tool [24]	Physicians and pharmacists	-	Authors expect to save 3 or more minutes per discharge prescription	There is uncertain about the support of the users with the tool	-
MedRec user interface [25]	Pharmacists, nurses, physicians and patients	-	Patient safety is expected to improve if the medication list is ordered alphabetically or by patient adherence to its medication	-	-
Twinlist [26, 35]	Physicians and pharmacists	-	Reduction in time to carry out the MedRec process with fewer clicks and scrolls	Generally, participants agreed that Twinlistwas clear and helpful	Reduction in multi-step animation after learning to use the tool
The RightRx Project [27, 36]	Physicians and pharmacists	At admission: 88.4% (pharmacists) 20.2%(physicians)and 13%(pharmacy students) At discharge: 96.1%(physicians) 74.7%(pharmacists)	Reduction in time to carry out the MedRecwith fewer scrolls	-	Incorporating order sentences about dose-based prescribing Giving access to the medication changes made during hospitalization
MedRec-web app [28]	Physicians and pharmacists	52%(general practitioners) 37.5%(physician specialist) 10.4% (pharmacists)	-	73% and 75% of participants considered, respectively, the medium prototype and high-fidelity prototype as acceptable	Integration with the ordering process Enhancing the interoperability with EMR and the edition of the patient medication list Color codes to highlight information and to alternate medications Reduction in the number of clicks
The Ambulatory electronic health record evaluation tool [29]	Physicians and pharmacists	Compliance rate 43%	Patient safety is expected to improve due to the access of users to the most updated patient medication list through the tool	Poorly understood by most of users	Training sessions Redesigning the app to facilitate the understandability of the tool Interoperability of EMRs beyond healthcare systems to avoid data entry errors
MedTrue [30]	Pharmacists, nurses and physicians	Users have not used MedTrue consistently	A more accurate picture of patient medication	Unsatisfied with MedTrue, giving it a rate of 0 out of 10	Integration into clinician workflow Reduction in the number of clicks

*PAML* Preadmission Medication List, *MedRec* Medication Reconciliation, *ADEs* Adverse drug events, *EMR* Electronical medical records

## Discussion

The results of the systematic review showed that 12 e-MedRec tools aimed at health professionals have been developed to date, 10 of them were software and only two were websites.A considerable number of e-tools were developed over the past five years, which could be justified by the increasing evidence about the beneficial role of using IT during the MedRec process [7, 10, 36]. Some of the e-tools presented innovative functionalities, for example, the generation of a conciliation report or alerting users about allergies/drug interactions. Users evaluated positively most e-MedRec tools in terms of adherence, usability, and satisfaction. The incorporation of “user-friendly” information and integration of the e-tools with the ordering process were the suggestions more frequently requested by users. The clinical impact of e-MedRec tools was achieved with the use of four e-tools in terms of reductions in adverse drug events, medication discrepancies, and medication errors, although no significant differences were found in other relevant outcomes such as emergency visits or hospital readmissions.

In spite of the high number of available e-health tools, there is very little evidence about e-MedRec tools. A previous review [9] about this issue highlighted that more studies are required to increase the knowledge about e-health in order to develop a sophisticated e-MedRec tool. Since the publication of that review [9], “Twinlist” [25], one of the e-tools mentioned, was analyzed in a subsequent study reporting promising results in terms of usability, satisfaction, and clinical impact on MedRec [31]. In addition to this, new four e-tools were developed [26–29]. Authors of the “RightRx Project” [26] designed their tool to focus on the needs of clinicians and to develop easily in order to improve the implementation, safety, and efficiency of the tool. The study which reports the “MedRec web-app” [27] revealed that a previous usability assessment of the e-tool is essential to perform a larger study evaluating its impact on clinical outcomes.On the contrary, “The Ambulatory electronic health record evaluation tool” [28] and “MedTrue” [29] received a low score in satisfaction surveys, so some changes may be needed in the next prototypes to maximize their functionalities compared to prior e-tools.

In the last decade, evolving support for the integration of MedRec apps with EMRs is emerging, such as the “Fast Health Interoperability Resources” (HL7-FHIR) [37], a platform for healthcare data exchange that could serve as a guide to developers about sources for medication information, availability of the data for providers and functional MedRec modules that a valuable MedRec tool should include. In addition, some authors have remarked that the social knowledge networking system could be useful to exchange routine issues related to EMR-MedRec with other professionals, to promote the creation of “learning healthcare systems” across provider subgroups and care settings [38]. These approaches may provide better ways of integrating efficient MedRec into clinical workflows and consequently, improving the quality of new prototypes of MedRec tools.

There is a wide heterogeneity of opinions about the ideal characteristics that e-MedRec tools should include.In fact, some clinicians have suggested the incorporation of some indispensable functionalities into health IT in order to facilitate their integration into clinician workflow [39]. In this sense, transferring information to other clinicians, integration into EMR systems, user-friendly information to minimize clinician workload and offering training sessions were some of the features more demanded to be included in e-health tools [40, 41]. Some authors also noted the need for interaction checkers among patient medication lists and herbal medicines and reminder alerts to users about allergy-causing medication [42]. None of the e-tools included in this systematic review compiled all of these items, but some users' suggestions agree on incorporating technical support [28], interoperability [19, 27], or saving time on the MedRec process [29]. Consequently, it would be desirable to establish comprehensive and reliable assessment criteria in this field [43], so it could enhance the design of more sophisticated e-MedRec tools shortly.

According to our results, none of the e-MedRec tools was designed as a mobile app, which contrasts with the wide range of disciplines covered by e-Health apps [44]. Recently, a descriptive study about health professionals managing drugs-related apps at emergency rooms revealed that only one of the 47 identified apps provided information related to medication reconciliation [8]. It consisted of a Spanish-language app that is available on iOS and Android platforms (https://en.apkbe.com/app/com.sefh.conciliacion) and included information about medications to reconcile in less than four hours since hospital admission, a browser of medication by therapeutic groups or the possibility to save relevant notes by users. Nevertheless, the quality assessment of the app was not explored yet, which reflected the lack of a comprehensive evaluation of e-MedRec tools noted by some authors [8, 9]. Patient security and privacy, ease of use and usage, time-consuming to manage the e-tool, cost, knowledge of e-health technology, communication between healthcare providers and patients, design, and technical support are some of the barriers that could explain the low percentage of apps detected [8, 36, 45–47].

Medication management e-tools have been mainly focused on self-patient care. However, healthcare professionals were rarely the target of available e-MedRec tools, despite performing an essential role during the MedRec process [9]. This finding could be due to the high variability of acceptance between clinicians of using IT in clinical practice [48]. Non-previous experience with IT, lack of training, and workload were some of the impediments cited by healthcare professionals to reject the use of IT [49, 50]. Additionally, some clinicians are still not aware of the impact of using an e-MedRec tool on clinical outcomes because of its low implementation in healthcare organizations [7]. In this sense, more efforts should be made to demonstrate to healthcare professionals the importance of incorporating e-tools into clinical practice.

The main limitation of this work is that iOS and Android platforms were not explored to identify e-MedRec tools, but only one app was detected in a previous study [8]. However,the search strategy was performed in four healthcare databases, which included a high number of studies from the highest impact journals, with no language restrictions or limitations in date publication. We also sent some questions by e-mail to all corresponding authors to compile more data about e-tools already reported with scarce information about their functionalities or clinical outcomes. Only three of them responded, and one author provided more detailed information about the future steps of the tool. Finally, further studies are needed to increase the limited evidence on e-MedRec tools, especially those designed as mobile apps, and to assess their clinical impact on patient safety.

## Conclusion

In conclusion, 12 e-MedRec tools aimed at health professionals have been developed to date, 10 of them were software and only two were websites. None of the e-MedRec tools was designed as a mobile app. The main features that healthcare professionals requested to be included in e-MedRec tools were interoperability, “user-friendly” information, and integration into the ordering process. Further studies would be needed to standardize the quality assessment of the e-tools and to evaluate rigorously their clinical impact on patient safety in the near future.

## Supplementary Information

Below is the link to the electronic supplementary material.

Supplementary file1 (DOCX 19 KB)

Supplementary file2 (DOCX 29 KB)

## Data Availability

Not applicable.
