# Supplementary material for: Electronic Medication Reconciliation Tools Aimed at Healthcare Professionals to Support Medication Reconciliation: a Systematic Review
Source: J Med Syst. 2023 Dec 6;48(1):2. doi: 10.1007/s10916-023-02008-0 (PMC10700201; doi:10.1007/s10916-023-02008-0)
Supplement: Supplementary file 1 — Supplementary file1 (DOCX 19 KB) [file 10916_2023_2008_MOESM1_ESM.docx]

**Electronic medication reconciliation tools aimed at healthcare professionals to support medication reconciliation: a systematic review**

**Authors: Pablo Ciudad-Gutiérrez^1^, Paula del Valle-Moreno^1^, Santiago José Lora-Escobar^1^, Ana Belén Guisado-Gil^1^, Eva Rocío Alfaro-Lara^1^.**

^1^Department of Pharmacy, University Hospital Virgen del Rocio, Seville, Spain

Corresponding author: Ana Belén Guisado-Gil. Department of Pharmacy, University Hospital Virgen del Rocio. Av. Manuel Siurot s/n. 41013 Seville, Spain. Email address: [anaguigil@gmail.com](mailto:anaguigil@gmail.com)

Table S1. Complete search strategy for different databases.

| HealthcareDatabases | Searchstrategy |
| --- | --- |
| PubMed | ("medical records systems, computerized"[MeSH Terms] OR "electronic health records"[MeSH Terms] OR "wireless technology"[MeSH Terms] OR "user computer interface"[MeSH Terms] OR "text messaging"[MeSH Terms] OR "electronic*"[Title/Abstract] OR "digital"[Title/Abstract] OR "mobile"[Title/Abstract] OR "user computer interface"[Title/Abstract] OR "medical record*"[Title/Abstract] OR "information technolog*"[Title/Abstract]) AND ("medication discrepancies"[Title/Abstract] OR "reconciliation discrepancies"[Title/Abstract] OR "medication reconciliation"[MeSH Terms] OR "medication reconciliation"[Title/Abstract]) |
| EMBASE | ('reconciliation discrepancies':ab,ti OR 'medication therapy management':ab,ti OR 'information technology':ab,ti OR 'computer interface':ab,ti OR 'wireless communication'/exp OR 'computer interface'/exp OR 'medication therapy management'/exp OR 'electronic health record'/exp) AND 'electronic medication reconciliation':ab,ti |
| Cochrane Library | reconciliation discrepancies:ti,ab,kw OR medication reconciliation:ti,ab,kw AND information technolog*:ti,ab,kw AND medical record*:ti,ab,kw AND user computer interface:ti,ab,kw AND electronic* health record*:ti,ab,kw |
| SCOPUS | TITLE-ABS-KEY medication OR reconciliation OR electronic* health record* OR medical record* OR user computer interface AND medication |
